# Early migration of stemless and stemmed humeral components after total shoulder arthroplasty for osteoarthritis—study protocol for a randomized controlled trial

**DOI:** 10.1186/s13063-020-04763-8

**Published:** 2020-10-07

**Authors:** Marc Randall Kristensen Nyring, Bo S. Olsen, Müjgan Yilmaz, Michael M. Petersen, Gunnar Flivik, Jeppe V. Rasmussen

**Affiliations:** 1 Department of Orthopedic Surgery, Herlev and Gentofte Hospital, University of Copenhagen, Copenhagen, Denmark; 2 Department of Orthopedic Surgery, Rigshospitalet, Copenhagen, Denmark; 3 Department of Clinical Medicine, University of Copenhagen, Copenhagen, Denmark; 4 Department of Orthopedic Surgery, Skaane University Hospital, Lund, Sweden

**Keywords:** Glenohumeral osteoarthritis, Stemless shoulder arthroplasty, Stemmed shoulder arthroplasty, Radiostereometric analysis, Shoulder replacement, Shoulder prosthesis

## Abstract

**Background:**

Glenohumeral osteoarthritis can, in the most severe cases, require surgery with insertion of a shoulder arthroplasty. A design with a stem in the humeral bone canal is currently regarded as the standard treatment option in patients who have an intact rotator cuff function, but complications related to the stem including humeral fractures can have devastating consequences. By using a stemless humeral component, stem-related complications can be reduced. The aim of this study is to compare the Comprehensive Nano stemless total shoulder arthroplasty (intervention group) with the Comprehensive stemmed total shoulder arthroplasty (control group).

**Materials and methods:**

This is a randomized controlled trial comparing the stemless and the stemmed total shoulder arthroplasty. All Danish citizens with glenohumeral osteoarthritis indicating a total shoulder arthroplasty referred to the orthopedic department at Copenhagen University Hospital in Herlev/Gentofte will be offered participation. The following exclude from participation: below 18 years of age, cognitive or linguistic impairment, insufficient function of the rotator cuff, poor bone quality, and ASA groups 4–5. A total of 122 patients will be included of which 56 will be part of a radiostereometric analysis (RSA) study of humeral component migration. The primary outcomes are magnitude of migration of the humeral component assessed by RSA and patient-reported outcome by Western Ontario Osteoarthritis of the Shoulder index (WOOS). The secondary outcomes are additional patient-reported outcomes, functional outcome, readmission, complications, revisions, and changes in bone mineral density (BMD) of the proximal humerus assessed by duel energy x-ray absorptiometry (DXA) and economy (cost-utility analysis). The patients are examined before the operation and 3, 6, 12, and 24 months postoperative.

**Discussion:**

To our knowledge, RSA has never been used to access migration of a stemmed or a stemless humeral component nor has the stemmed and the stemless humeral component been compared with regard to pain relief and shoulder function in a randomized clinical trial. Today, the two designs are considered equal in the treatment of osteoarthritis. The study will provide surgeons and patients with information about shoulder arthroplasty for osteoarthritis and assist them in decision-making.

**Trial registration:**

ClinicalTrials.gov NCT04105478. Registered on 25 September 2019

## Background

Previous literature has shown that anatomical total shoulder arthroplasty (an artificial shoulder joint with replacement of both the humeral head and the glenoid cavity) is an effective treatment of end-stage glenohumeral osteoarthritis with pain relief and significant improvement in shoulder function [1–4]. The outcome is better than the outcome of hemiarthroplasty (an artificial shoulder joint with replacement of only the humeral head), and it is recommended by the American Academy of Orthopedic Surgeons in patients with painful osteoarthritis and an intact rotator cuff function [5]. A stemmed humeral component applied either with or without cement is commonly used and is currently considered to be the gold standard. Nevertheless, complications related to the stemmed humeral component can have devastating consequences [6].

The prevalence of periprosthetic humeral fractures after total shoulder arthroplasty is between 1.6 and 2.4% [6]. These patients can be treated either non-surgically with risk of non-union or mal-union or they can be treated surgically, which, in most cases, is technically demanding. Removal of a well-fixed humeral stem is difficult and may result in additional fracture or destruction of the humeral bone and the outcome is often disappointing [7–10].

The stemless humeral component is fixed only in the metaphysis (the wide portion of the long bones where bone growth occurs in childhood). By using this stemless metaphyseal fixation, the risk of a periprosthetic fracture and other stem-related complications can be reduced. There are also other advantages of the stemless design [11]. When using a stemmed humeral component, the inclination (the angle between the head and the shaft of the humerus) is given in advance. Even though the modern designs have variable inclination options to choose from, it can be difficult to restore the anatomy. With preoperative planning and precise cutting instrumentation of the stemless humeral component, the individual anatomy can, in theory, be more easily restored. In addition, the stemless design may be associated with shorter operation time compared with the stemmed design. Finally, because of the canal preserving design, the revision procedure is facilitated should the need of a revision arthroplasty arise, making the stemless design easier revisable. However, this is probably not as important as it used to be, since most modern stemmed designs have a common platform system which allows the stem to be retained in the case of revision to a reverse shoulder arthroplasty due to rotator cuff problems. The theoretical advantages of the stemless design may lead to a superior functional outcome compared with the stemmed total shoulder arthroplasty.

However, the stemless humeral component relies extensively on good metaphyseal bone quality, and the risk of osteolysis, stress shielding, and aseptic loosening may be higher than for the stemmed humeral component with peripheral fixation where the bone quality and density is superior to the central bone [12, 13]. This may lead to a higher risk of migration of the component.

Plain radiographs are unable to detect minor implant migration, and authors have recommended that radiostereometric analysis (RSA) should be used instead [14, 15]. By inserting small tantalum beads (0.8 mm) on the arthroplasty component and into the surrounding bone, implant migration can be measured extremely accurately [16]. Technical advances within RSA have made it possible to identify the implant and its position using the geometry of the implant instead of attaching tantalum beads to the implant (model-based radiostereometric analysis, MB-RSA). The MB-RSA technique is less precise than the marker-based method. However, precision error values are still acceptable for clinical studies aimed at evaluating implant migration [17]. The use of RSA for evaluation of implant migration has been used frequently in the evaluation of hip and knee arthroplasty surgery. It has been shown that the trend for late implant aseptic loosening of the tibial component in total knee arthroplasty is consistent with early RSA findings of continuous migration past the first postoperative year [18]. Therefore, it has been suggested that a small series of a new arthroplasty should be monitored with RSA the first 2 years postoperatively, as a part of a safe phased introduction of new arthroplasties [19–21].

Changes in bone mineral density (BMD) in close relation to an orthopedic implant can be measured by dual-energy x-ray absorptiometry (DXA) [22, 23]. The use of DXA for prospective quantitative evaluation of implant induced adaptive bone remodeling has been widely used after both hip and knee primary replacement surgery [24–28]. Only few studies [29, 30] have evaluated changes in BMD after shoulder arthroplasty. After an initial decrease, probably caused by postoperative immobilization [31], BMD of the proximal humerus increased and was higher than the initial value at follow-up 1 and 2 years postoperatively [30].

In shoulder surgery, RSA has been used to study the migration of the glenoid component [32, 33] and, in few cases, migration of hydroxy-coated resurfacing humeral components [30, 34]. To our knowledge, RSA has never been used to access migration of a stemmed or a stemless humeral component nor has the stemmed and the stemless humeral component been compared in a randomized design with regard to clinical outcome and patient-reported outcome.

The aim of this study is to compare the Comprehensive Nano stemless total shoulder arthroplasty (intervention group) with the Comprehensive stemmed total shoulder arthroplasty (control group). Our hypotheses are:

1. The Comprehensive Nano stemless total shoulder arthroplasty will migrate more than the Comprehensive stemmed total shoulder arthroplasty.
2. The Comprehensive Nano stemless total shoulder arthroplasty will have a superior functional outcome compared with the Comprehensive stemmed total shoulder arthroplasty.

## Methods and design

### Study design

This is an investigator-initiated, single-center, 1:1 randomized controlled, superiority trial with parallel groups, comparing the Comprehensive Nano stemless total shoulder arthroplasty (intervention group) with the Comprehensive stemmed total shoulder arthroplasty (control group). Both implants are manufactured by Zimmer Biomet (Warsaw, IN, USA).

### Method

Inclusion criteria:

1. Primary glenohumeral osteoarthritis independent of previous joint preserving surgery
2. Osteoarthritis on plain radiographs with standard anterior-posterior and lateral projections
3. Insufficient effect of non-surgical treatment with symptoms severe enough to justify shoulder arthroplasty.
4. ASA scores 1–3, physically fit for surgery and rehabilitation

Exclusion criteria:

1. Below 18 years of age
2. Cognitive or linguistic impairment
3. Rotator cuff insufficiency defined as rotator cuff lesions or grade 2 fat infiltrations on MRI according to the Goutallier classification [35, 36] verified with impaired functional strength and perioperative findings
4. Insufficient glenoid bone-stock or large (> 1 cm) humeral bone cysts on CT verified with perioperative findings
5. ASA scores 4–5

### Enrolment

**Fig. 1:**
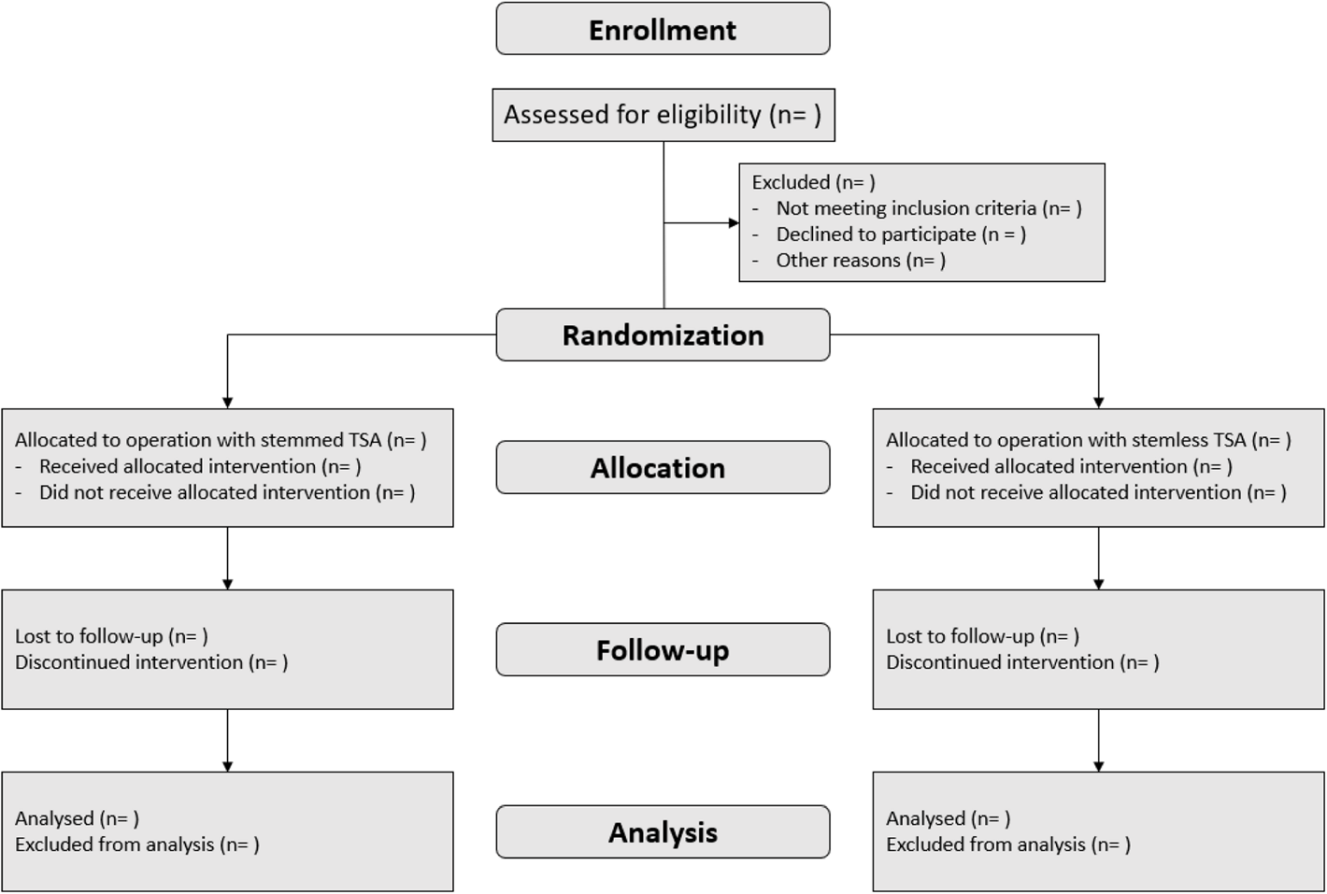
CONSORT flow diagram. The expected flow of patients through the study

All Danish citizens with glenohumeral osteoarthritis indicating an anatomical total shoulder arthroplasty referred to the orthopedic department at Copenhagen University Hospital in Herlev/Gentofte will be considered for participation in the trial. The treating physician will review the medical records and assess whether the patient fulfills the above-mentioned inclusion criteria and none of the following exclusion criteria. If so, the treating physician will pass on the information to the responsible investigator (Fig. 1) and the patient will be offered participation.

The patients will be called in for an undisturbed consultation with the treating physician. If the treating physician considers the patient suitable for inclusion in the project, the patient will receive oral and written information regarding the study. Furthermore, they will be informed about the possibility to bring a bystander for the preparation consultation with the corresponding investigator. The preparation consultation with the corresponding investigator will then be held at least 24 h later in an undisturbed ambulatory room. At this consultation, the patient (and a possible bystander) will once again receive information regarding the study and subsequently the informed consent will be obtained. The patient has thus been given at least 24 h of reflection time, as well as being given the opportunity to bring a bystander.

The informed consent will give the primary investigator access to information about comorbidity, medication, education, occupation, age, and gender. The patients will then be asked to complete 4 questionnaires (Oxford Shoulder Score, Western Ontario Osteoarthritis of the Shoulder index, EQ-5D-5L, and pain). They will also have a clinical examination with measurement of pain, activity of daily living, range of movement, and strength (Constant-Murley score) and a radiographic examination with plain radiographs with standard anterior-posterior and lateral projections, CT scan, and MRI of the index shoulder joint. Finally, DXA is used to evaluate osteopenia and BMD of the proximal humerus and distal forearm. There are no plans of additional studies or use of data beyond this trial.

### Randomization

Based on the sample calculation, we intend to include a total number of 122 patients of which the first 56 patients who agree to participate will be studied using MB-RSA and DXA. The patients are allocated into two groups of equal of size and equally randomized to both arms:

1. The uncemented Comprehensive Nano stemless shoulder arthroplasty (intervention group)
2. The uncemented Comprehensive stemmed total shoulder arthroplasty (control group)

The randomization is done in the operating theater using the trial laptop just after the perioperative evaluation of the bone-stock and the rotator cuff. Therefore, the patient is anesthetized when the randomization is done. The randomization is done using a computerized irreversible application—the Research Electronic Data Capture (REdCap). The randomization sequence will be computer generated by block randomization stratified for age and gender. The patients are equally randomized to both arms (1:1 allocation). The randomization table has been prepared by an independent statistician.

The patients and the outcome assessor will not be blinded to the randomization allocation. However, the statistician associated with the trial will be blinded.

### Surgical procedure and rehabilitation

All procedures are performed at Copenhagen University Hospital Herlev/Gentofte, which is the largest shoulder arthroplasty unit in Denmark with approximately 250 primary and revision shoulder arthroplasty procedures each year. Based on the number of patients from 2013 to 2016, we expect to operate 50 patients with an anatomical total shoulder arthroplasty for osteoarthritis each year. In order to secure a high surgical standard and to minimize a potential learning curve, the study will be conducted as a single-center study and all patients will be operated on by one of six senior consultant shoulder surgeons with many years of experience and hundreds of shoulder arthroplasties on their resumes. Prior to the initiation of the study, each of the surgeons should have performed at least 3 procedures with the Comprehensive Nano stemless total shoulder arthroplasty. A subgroup analysis, comparing the first half of patients to be included with the second half, is used to discuss the learning curve of the stemless humeral component.

Surgical technique, including exposure instrumentation and soft tissue balancing, is standardized for all patients. The procedure is performed with the patient under general anesthesia in beach chair position. All patients are operated on with the standard deltopectoral approach and subscapularis tenodesis. The patients are treated with either (1) the uncemented Comprehensive Nano humeral component or (2) the uncemented stemmed Comprehensive humeral component according to the guidelines from the manufacturer. The uncemented Comprehensive Nano humeral component is a star-shaped component made of titanium and coated with porous plasma spray. The uncemented stemmed Comprehensive humeral component has a standard stem length of 122 mm and is made of titanium with proximal porous plasma spray coating. A standard Comprehensive Modular Hybrid Glenoid component (Zimmer Biomet, Warsaw, IN, USA) is used in all patients. It is made of polyethylene and consists of three peripheral polyethylene pegs inserted with bone cement and one central titanium peg inserted without bone cement. The humeral bone component is marked with 8–10 RSA markers inserted by the surgeon. Prophylactic cloxacillin 2 g and benzylpenicillin 1.2 g is given preoperative and at 6 and 12 h. If a patient cannot tolerate the standard prophylactic treatment, cefuroxime 1.5 g is given preoperative and at 6 and 12 h. No biological samples will be collected.

All patients have been encouraged to perform non-operative treatment with training, painkillers, possible cortisone injections, and rescheduling of work and leisure activities. If there is no effect of these preoperative treatment attempts, the indication for surgical treatment is given by one of the six senior consultant shoulder surgeons.

Postoperatively, a simple sling will be used for 2 weeks. Before discharge, the patients are instructed to perform edema prophylaxis during the time of immobilization. All patients will follow a standard rehabilitation program supervised by a physiotherapist once a week. Non-weight bearing training is allowed after 2 weeks without any restriction on active external rotation. Weight bearing training is allowed after 6 weeks. There is a minimum of 3 months of training, longer if needed. The patients are allowed to participate in additional rehabilitation if they wish to do so.

### Outcome measures

#### Primary radiological outcome

##### Magnitude of migration of the humeral component assessed by MB-RSA

The MB-RSA will be performed according to the guidelines by Valstar and Colleagues [15]. RSA is used to measure humeral component migration. Fifty-six patients will be included in this part of the study. Maximum total point motion (MTPM) after 2 years compared to the baseline value will be used as the primary RSA effect parameter. The precision of RSA will be evaluated by at least 12 double examinations. RSA is performed using a uniplanar RSA arrangement (UmRSA®-Calibration Cage No 43 (hip, spine, and shoulder)). The analysis of x-rays will be performed using the MB-RSA commercial software (RSAcore, Department of Orthopedics, Leiden University Medical Center, Leiden, The Netherlands), available at Skaane University Hospital, Lund Sweden, where analyses of RSA-x-rays will be performed using a well-established research cooperation with consultant orthopedic surgeon, PhD, Gunnar Flivik. The precise set-up for the RSA arrangement (e.g., various distances and the degree between the 2 x-ray tubes) will be determined from a small phantom study and measurements of the 6 pilot patients (3 with each of the 2 types of implants). In addition, the pattern of migration of the humeral component assessed by MB-RSA will be presented using descriptive statistics, but without the performance of statistical tests.

#### Primary functional outcome

##### Western Ontario Osteoarthritis of the Shoulder index (WOOS)

The WOOS is a disease-specific patient-reported outcome [37]. There are 19 questions divided into four domains: physical symptoms, sports and work, lifestyle, and emotions. Each question is answered on a visual analogue scale ranging from 0 to 100. The overall score ranges from 0 to 1900, with 1900 being the worst. For ease of interpretation, the scores are converted to a percentage of the maximum score. The primary value of interest is the difference in mean score between the preoperative score and 2-year follow-up. We use a Danish version of WOOS which was translated according to the guidelines of Guillemin, Bombardier, and Beaton [38]. It was validated with classical test theory in a cohort of patients treated with shoulder arthroplasty for osteoarthritis [39]. No minimal clinically important difference for WOOS has been reported yet.

#### Secondary radiological outcomes

##### DXA

Fifty-six patients will be included in this part of the study. DXA will be performed preoperatively to evaluate BMD of the proximal humerus and distal forearm. Bilateral BMD measurements of the proximal humerus and distal forearm will be used to adjust for changes in BMD that are not related to the shoulder arthroplasty. The primary value of interest is the difference in mean BMD between the preoperative score and 2-year follow-up. The precision of DXA will be evaluated by double examinations.

##### Plain radiographs

The radiographs are taken preoperatively, within the first week after surgery, at 3 months, and at 1 and 2 years. We use an anterior-posterior and a lateral view. The radiographs are used to evaluate the position of the component and to evaluate loosening as the cause of failure.

#### Secondary functional outcomes

##### Oxford shoulder score (OSS)

The OSS was conceived as a measurement tool for the assessment of pain and function after elective shoulder surgery [40]. There are 12 questions with each item scored from 0 to 4. The overall score ranges from 0 to 48, with 48 being the best. For ease of interpretation, the scores are converted to a percentage of the maximum score. The primary value of interest is the difference in mean score between the preoperative score and 2-year follow-up. We use a Danish version of OSS which was translated and validated with classical test theory [41]. The minimal clinically important difference for OSS has been suggested to be 6 points [42].

##### Constant-Murley score

The Constant-Murley score includes an assessment of pain, activities of daily living (ADL), range of motion, and strength. There are a possible 35 points given for the subjective assessment of pain and the ability to perform ADL. There are a possible 65 points given for an objective assessment, of which 40 points are allocated to range of motion and 25 points are allocated to strength. The maximum of 100 points indicates a shoulder with no disability. The primary value of interest is the difference in mean score between the preoperative score and 2-year follow-up. We use a Danish version [43] of the modified score described by Constant and colleagues in 2008 [44].

##### Pain and patient satisfaction

Pain on the day of examination is answered on a visual analogue scale (VAS) ranging from 0 to 100, with 100 being the worst. Patients are asked to categorize the result on a 7-point scale ranging from much worse to much better. The primary value of interest is the difference in mean score between the preoperative score and 2-year follow-up.

##### Side effects and complications

We will record any case of medical complications (embolism, cardiovascular event, pneumonia) and complications related to the surgical procedure (fractures, nerve injuries, deep and superficial infections, malpositioning of the components, instability, and dislocation) and revisions defined by removal or exchange of any component.

##### Economic evaluation

In modern health economics, thresholds have been estimated for acceptable cost-utility ratios—the threshold for how much health care providers will pay for an extra quality-adjusted life year (QALY). In England and in Europe, the threshold for an extra QALY is set at 20,000–30,000 pounds and 30,000 Euros, respectively. The cost utility of the Comprehensive Nano stemless shoulder arthroplasty will be compared with these thresholds and with the cost utility of the uncemented Comprehensive stemmed total shoulder arthroplasty. The EQ-5D-5L will be used to estimate QALY for individual patients. The primary value of interest is the difference in mean score between the preoperative score and 2-year follow-up. A cost model will be defined using data from patients, clinical records, registries, and unit costs from the Danish health care system. We will record length of hospital stay, discharge destination, pain medication usage, and readmission.

### Follow-up

**Fig. 2:**
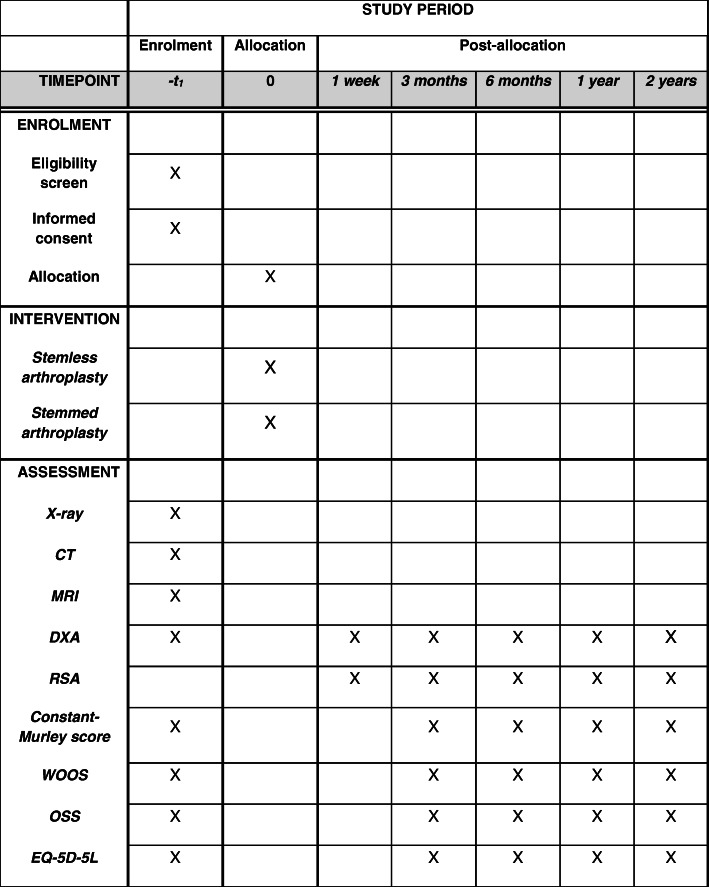
SPIRIT figure. Schedule of enrolment, interventions, and assessments of the 56 RSA patients

**Fig. 3:**
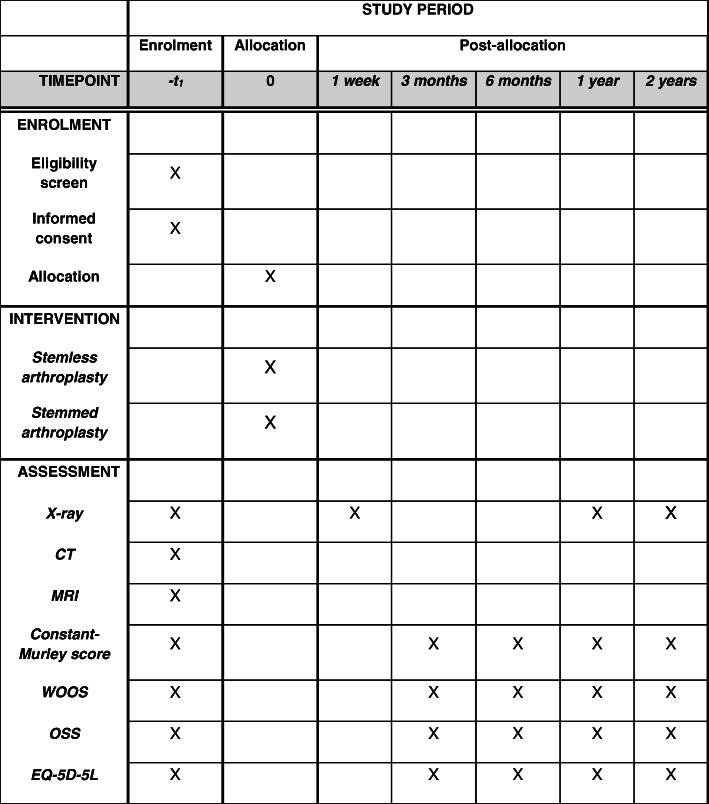
SPIRIT figure. Schedule of enrolment, interventions, and assessments of the 66 patients who are not a part of the RSA study

All patients will be followed actively for 2 years (Figs. 2 and 3) with evaluation at 3, 6, 12, and 24 months from the day of surgery (and randomization). Data on complications including revision will be extracted from the hospital database and patient records after 10 years.

### Protocol violations, patient drop-out, and revision

If a stemless shoulder arthroplasty during the operation is regarded impossible to insert by the surgeon, the operation can be converted to either cemented or uncemented stemmed total shoulder arthroplasty or cemented or uncemented reversed shoulder arthroplasty. The uncemented stemmed shoulder arthroplasty can be converted to either cemented stemmed total shoulder arthroplasty or cemented reversed shoulder arthroplasty. The patient will be excluded according to the exclusion criteria, and the conversion will be recorded. If the conversion is performed after the randomization, then the patient will be excluded from the RSA and DXA analysis but included in the analysis of the functional outcome and the patient-reported outcome (intention-to-treat).

Patients who drop-out of the trial will be recorded, and the reason for drop-out will be noted. The patient will be included in the final mixed effects model analysis. Drop-outs before the 3-month evaluation cannot be included in the analysis. In addition, a sensitivity analysis will be carried out at the end of the study to assess the impact of missing data on the overall trial results. Patients who do not comply with rehabilitation will be recorded.

If a revision arthroplasty is needed, the reason and the new arthroplasty type are recorded. If possible, the patient is evaluated using RSA, DXA, clinical outcome, and patient-reported outcome prior to the revision procedure. The patient will remain in the study, and they are included in the 2-year analysis with their latest follow-up.

Major protocol modifications will be communicated to all investigators and trial registries immediately.

### Statistics

The nature of data is evaluated before the analysis. The between-group differences are expected to be analyzed with independent *t* test (Student *t* test). If the assumption of normal distribution is violated, then data will be analyzed with non-parametric tests. We do not expect the RSA data to be normal distributed. These data will be analyzed with non-parametric analysis using the Friedmann test for migration over time and the Mann-Whitney *U* test for between-group differences. We expect the DXA data to be normally distributed and changes over time will be evaluated by a parametric analysis using a paired *t* test. Potential between-group differences in BMD changes will be analyzed with independent *t* test (Student *t* test). In addition, due to the nature of repeated measures in this study, we intend to analyze the overall results according to a mixed effects model [45]. The difference between pre- and postoperative scores is expected to be analyzed as a secondary endpoint with parametric analysis using a paired *t* test. Since the two primary endpoints are distinct, multiple testing adjustments will not be performed.

#### Sample size calculation RSA

Sample size calculation for RSA (maximum total point motion (MTPM) after 2 years will be used as primary RSA effect parameter) with an expected standard deviation (SD) of 0.4 mm, a minimally clinically important difference of 0.4 mm, a significance level at 5%, and a power of 0.90 resulted in 22 participants in each group [46] (56 participants with allowance for 20% drop-out). We intend to include 28 participants in each group. However, the study entails a risk of drop-out and unwillingness to participate, but according to the power calculation, we would be satisfied with a minimum of 22 participants in each group. The estimation of SD = 0.4 mm is extrapolated from a study of migration of the resurfacing arthroplasty [34].

#### Sample size calculation WOOS

With a standard deviation of WOOS of 15 (15% of a maximum score), a difference of 10 (10% of a maximum score), a significance level of 5%, and a power of 0.90, 48 patients are needed in each group resulting in a study population with 122 participants [46] (allowing for 20% drop-out). No minimally clinically important difference of the WOOS score has ever been reported. Therefore, it is important to mention that the use of 10 points in this study is an arbitrary value.

Data on RSA and DXA will be analyzed at the end of follow-up for 56 patients. We have chosen not to perform a 1-year formalized interim analysis, but an external independent assessor from another hospital will once a year get an update on the study and review the results in a confidential manner. In this way, the scientific validity of the study will be improved.

## Discussion

Apart from the humeral component, the two groups are treated alike. Today, the Comprehensive Nano stemless humeral component and the uncemented Comprehensive stemmed humeral component are considered equal in the treatment of osteoarthritis [47].

We intend to decrease the risk of selection bias through block randomization stratified for age and gender. Through sample size calculation and adding 20% to the result, we compensate for drop-outs and attempt to avoid type II errors (when the null hypothesis is false but fails to be rejected).

The patients in both groups will follow the standard rehabilitation program supervised by a physiotherapist, and furthermore, the surgical approach and technique will follow the standard procedures and be equal in the two groups. This leads to a high external validity of the study. The standard treatment at our department includes a follow-up examination at 3 months. The participating patients will have additional follow-up examinations at 6 months and at 1 and 2 years. Besides being time consuming, this may maintain the patients in a role of being ill. However, the patients will also have the advantage of feeling secure and any uncertainty or problems can be addressed more easily.

With this study, all patients are at risk of being treated with a component that subsequent analyses will deem inferior. This is implicit in the study design, but there is nothing a priori to suggest which technique is the better.

This is a single-center study with all operations being carried out by one of six highly experienced shoulder surgeons. Therefore, the results of the study can only be transferred directly to patients operated by surgeons with the same skills. Thus, we acknowledge that this nature of the study entails a risk of less generalizability.

The patients will not be blinded to their randomization allocation. This would be very difficult in Denmark, as patients have access to their records electronically. However, this is not expected to be of great significance as the patients hardly have an expectation of one of the prostheses to be the superior. Furthermore, it is not possible to blind the outcome assessor as it is the same person analyzing the RSA and DXA, in which one must necessarily have access to the radiographs. However, this is not expected to be of any significance to the second primary outcome, since this is a patient-reported outcome which is completed by the patient independently of the outcome assessor. In addition, the final analyses will be carried out by a blinded statistician associated with the trial.

In a study based on data from the Nordic Arthroplasty Register Association [47], the total stemless shoulder arthroplasty and the total stemmed shoulder arthroplasty appear to have comparable short-time survival. However, it is not known whether they continue to perform equally. Ryd et al. [18] and Kärrholm et al. [48] both found a relationship between short-term RSA results and future loosening of prostheses. They found that already 1-year postoperative RSA can be used to identify prostheses at risk of future loosening. Therefore, the use of RSA in this study will enable us to predict the long-term results of the two arthroplasties.

The main value of the study is new knowledge about the best treatment option for end-stage osteoarthritis. This can be of value to future patients with end-stage osteoarthritis where shoulder arthroplasty is considered.

### Trial status

Version 1.4, October 10, 2019.

Start of inclusion: March 1, 2020.

Finnish date of recruitment to the first part of the study (RSA and DXA): February 28, 2021.

Finnish date of recruitment of all 122 patients: March 31, 2022.

Finnish date of follow-up for the first part of the study (RSA and DXA): February 28, 2023.

Finnish date of follow-up for all 122 patients: March 31, 2024.

## Supplementary information

**Additional file 1.** Western Ontario Osteoarthritis of the Shoulder index (WOOS).

**Additional file 2.** Oxford shoulder score.

**Additional file 3.** Constant Score.

**Additional file 4.** External assessor.

**Additional file 5.** Informed consent to participate in the health science research project.

**Additional file 6.** Participant information.

**Additional file 7.** Data category and information.

**Additional file 8.** Justification for criteria left as N/A.

## Data Availability

The electronic data will be stored in closed drives and printed data will be stored in closed cabinets. The datasets used and/or analyzed during the current study are available from the corresponding author on reasonable request.

## Abbreviations

RSA: Radiostereometric analysis
BMD: Bone mineral density
DXA: Duel energy x-ray absorptiometry
MB-RSA: Model-based radiostereometric analysis
WOOS: Western Ontario Osteoarthritis of the Shoulder index
OSS: Oxford Shoulder Score
ADL: Activities of daily living
VAS: Visual analogue scale
QALY: Quality-adjusted life year
SD: Standard deviation
MTPM: Maximum total point motion
