# Supplementary material for: Early migration of stemless and stemmed humeral components after total shoulder arthroplasty for osteoarthritis—study protocol for a randomized controlled trial
Source: Trials. 2020 Oct 7;21:830. doi: 10.1186/s13063-020-04763-8 (PMC7541322; doi:10.1186/s13063-020-04763-8)
Supplement: Supplementary file 1 — Additional file 1. Western Ontario Osteoarthritis of the Shoulder index (WOOS). [file 13063_2020_4763_MOESM1_ESM.doc]

**Dansk oversættelse af:**

«cpr» «opdato»

«Navn»

«Adresse»

«PostNr» «By»


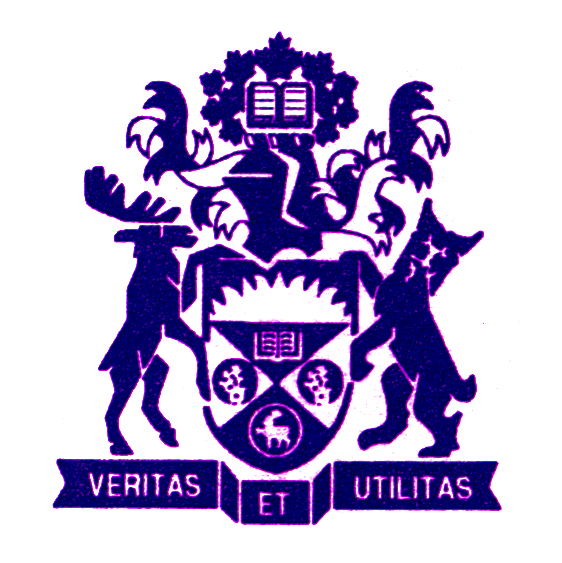


WESTERN ONTARIO

OSTEOARTHRITIS

OF THE SHOULDER

INDEX (WOOS)©

# Et redskab til måling af livskvalitet ved patienter med gigtlidelse

# eller kunstigt led i skulderen

Copyright © 1998, A. Kirkley, MD, S.Griffin CSS, I. Lo, MD

Oversættelse 2003, John Jakobsen, Ortopædkirurgisk Klinik, Aalborg

**PATIENTVEJLEDNING**

I det følgende spørgeskema vil du blive anmodet om at besvare spørgsmål i det følgende format, og du skal svare ved at sætte en skråstreg ”/” på den vandrette linie.

**Bemærk:**

1. Hvis du sætter en skråstreg “/” i den venstre ende af linien:

så angiver du, at du ikke oplever den angivne situation.

2. Hvis du sætter en skråstreg “/” i højre ende af linien:

 

så angiver du, at du oplever situationen I ekstrem grad.

3. Bemærk venligst:

a) at jo længere mod højre du anbringer skråstregen “/”, jo mere oplever du dette symptom.

b) at jo længere mod venstre du anbringer skråstregen ”/”, jo mindre oplever du dette symptomer.

**c) Sæt venligst ikke skråstregen “/” udenfor endemarkeringerne.**

I dette spørgeskema bliver du bedt om at angive graden af symptomer, du har følt i den sidste uge med hensyn til din problematiske skulder. Hvis du er usikker på, hvilken skulder der er involveret, eller du har andre spørgsmål, så spørg før du udfylder spørgeskemaet.

Hvis du af en eller anden grund ikke forstår et spørgsmål, så læs den forklaring som står i slutningen af spørgeskemaet. Du kan så sætte skråstregen “/” et passende sted på den vandrette linie. Hvis et spørgsmål ikke er relevant for dig eller du ikke har oplevet det i den seneste uge, så kom med dit bedste bud på, hvilket svar der ville være mest nøjagtigt.

**AFSNIT A: Fysiske symptomer**

**De følgende spørgsmål omhandler de fysiske symptomer, du har på grund af dit skulderproblem. Ved hvert spørgsmål skal du sætte en skråstreg ”/” på linien for at markere omfanget af dine symptomer indenfor den seneste uge.**

1. Hvor megen smerte føler du i din skulder, når du bevæger den?

ingen ekstrem

smerte smerte

2. Hvor megen konstant vedvarende smerte føler du i din skulder?

ingen ekstrem

smerte smerte

3. Hvor meget kraftnedsættelse føler du i din skulder?

ingen ekstrem

nedsættelse nedsættelse

4. Hvor megen stivhed føler du i din skulder?

ingen ekstrem

stivhed stivhed

5. Hvor meget skurren føler du i din skulder?

ingen ekstrem

6. Hvor meget er din skulder påvirket af vejret?

ikke ekstremt påvirket påvirket

**AFSNIT B: Sport/fritid/arbejde**

**Følgende afsnit handler om, hvor meget dit skulderproblem har påvirket dine sports- og fritidsaktiviteter indenfor den seneste uge. Du skal igen til hvert spørgsmål markere omfanget af dine symptomer med en skråstreg ”/”.**

7. Hvor meget besvær har du med at arbejde eller nå noget over skulderhøjde?

intet ekstremt

besvær besvær

8. Hvor meget besvær har du ved at løfte ting (fx. indkøbsposer, affaldsposer osv.) under skulderhøjde?

intet ekstremt

besvær besvær

9. Hvor meget besvær har du med at gøre gentagne bevægelser under skulderhøjde som fx. at rive, feje eller vaske gulv på grund af din skulder?

intet ekstremt

besvær besvær

10. Hvor meget besvær har du med at skubbe eller trække noget tungt på grund af din skulder?

intet ekstremt

besvær besvær

11. Hvor besværet er du af forværring af skuldersmerterne, når du har brugt den?

ikke ekstremt

besværet besværet

**AFSNIT C: Livsstil**

**Dette afsnit handler om, hvordan dit skulderproblem har påvirket eller ændret din livsstil. Marker omfanget som i de foregående afsnit med en skråstreg ”/”.**

12. Hvor meget besvær har du med at sove på grund af din skulder?

intet ekstremt

besvær besvær

13. Hvor meget besvær har du med at ordne dit hår på grund af skulderen?

intet ekstremt besvær besvær

14. Hvor meget besvær har du med at holde dig I form i det niveau du ønsker på grund af din skulder?

intet ekstremt

besvær besvær

15. Hvor meget besvær har du ved at række bagud og stoppe en skjorte ned, tage en pung fra baglommen eller ordne dit tøj?

intet ekstremt

besvær besvær

16. Hvor meget besvær har du med af- og påklædning på grund af din skulder?

intet ekstremt

besvær besvær

**AFSNIT D: Følelser**

**De følgende spørgsmål handler om, hvordan du har følt det den sidste uge med hensyn til dit skulderproblem. Marker omfanget med en skråstreg ”/”.**

17. Hvor megen frustration eller modløshed føler du på grund af din skulder?

ingen ekstrem

18. Hvor bekymret er du for, hvad der vil ske med din skulder i fremtiden?

slet ekstremt ikke bekymret bekymret

19. Hvor meget til besvær føler du, at du er for andre?

slet ekstremt

ikke meget

**Supplerende spørgsmål**

1. Hvis en ”normal” skulder fungerer 100%, hvordan fungerer din skulder så?

%

1.
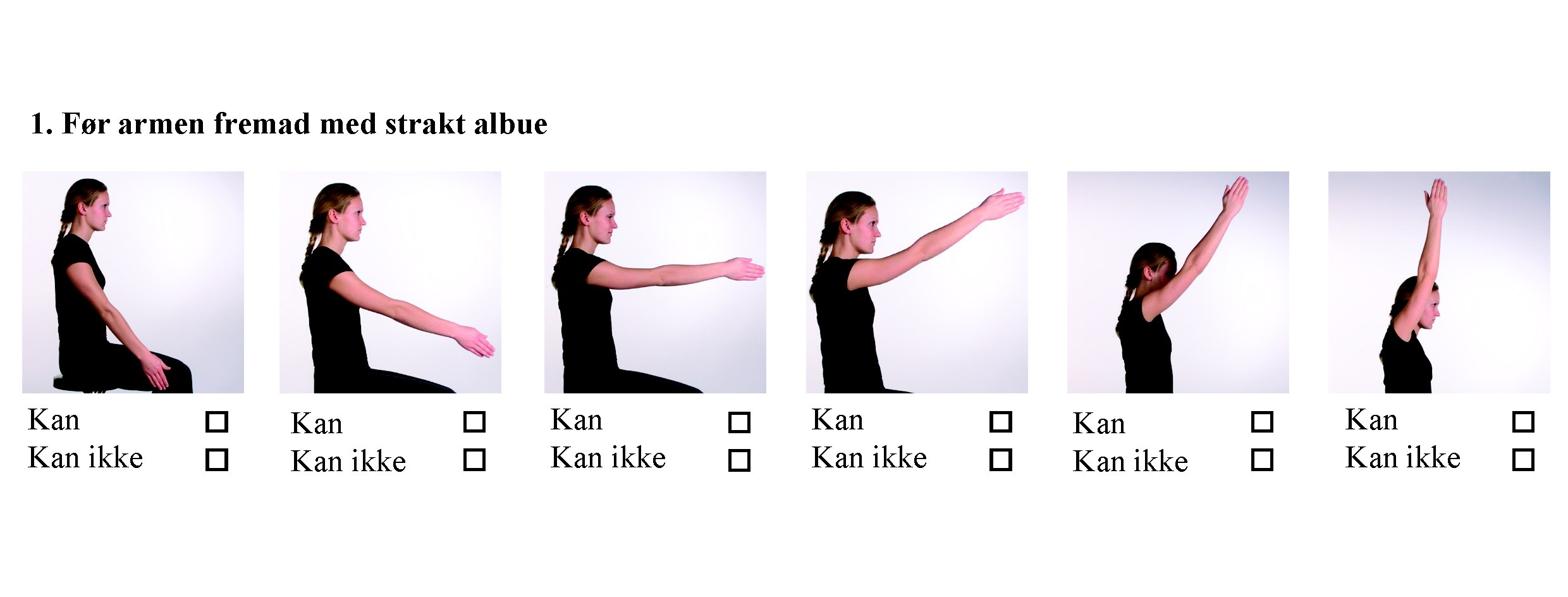
Marker ud for hvert billede om du med strakt albue kan løfte armen som vist

**TAK FORDI DU VILLE UDFYLDE SPØRGESKEMAET**

**Hvad betyder spørgsmålene?**

**Afsnit A: Fysiske symptomer**

Spørgsmål 1

Drejer sig om enhver pludselig jagende eller skarp smerte du føler, når du bevæger din arm.

Spørgsmål 2

Drejer sig om den murrende langvarige baggrundssmerte, som er ret konstant i modsætning til den pludselige skarpe smerte, der henføres til i forklaringen på spørgsmål 1.

Spørgsmål 3

Drejer sig om mangel på styrke til at udføre en bevægelse eller aktivitet.

Spørgsmål 4

Drejer sig om følelsen af , at din skulder ikke vil bevæges eller føles stiv eller låst. Dette kan føles om morgenen, når man står op eller efter en periode uden aktivitet.

Spørgsmål 5

Drejer sig om enhver lyd og/eller følelse du oplever i din skulder ved bevægelse, ofte beskrives det som skurren, knagen, knækken eller klikken.

Spørgsmål 6

Mange mennesker føler at smerter eller stivhed i skulderen ændrer sig med vejret. Nogle føler det ved en ændring i atmosfærisk tryk eller temperatur. Overvej venligst alle disse omstændigheder.

**Afsnit B: Sport/fritid/arbejde**

Spørgsmål 7

Drejer sig om enhver aktivitet som kræver, at du løfter din arm over skulderhøjde. Det kan være for at skifte en loftspære, pudse et vindue, nå en høj hylde, hænge tøj i skabet eller lægge et stort lagen sammen.

Spørgsmål 8

Dette drejer sig om at løfte ting i enhver højde under skulderen såsom en indkøbspose, bowlingkugle, sodavandskasse, affaldsspand, bøger, udstyr på arbejde eller at anbringe noget i ovnen.

Spørgsmål 9

Drejer sig om at gøre en frem- og tilbage- eller cirkel-bevægelse igen og igen under skulderhøjde.

Spørgsmål 10

Drejer sig om enhver aktivitet som kræver styrke i sin udførelse såsom at skubbe eller trække en tung støvsuger, åbne eller lukke en tung dør eller vindue, trække planter op af haven, tage strømper på, skifte gear i en bil eller flytte møbler.

Spørgsmål 11

Drejer sig om ekstra smerter du føler efter aktiviteter, som kræver brug af din skulder..

**Afsnit C: Livsstil**

Spørgsmål 12

Drejer sig om den virkning din skulder har på dine sædvanlige sovevaner såsom at blive nødt til at skifte sovestilling, at vågne om natten, ikke at kunne falde i søvn, have besvær med at ligge behageligt eller at vågne op med følelse af ikke at være udhvilet.

Spørgsmål 13

Drejer sig om alt du gør med dit hår såsom daglig frisering, børstning eller vask, som kræver, at du kan nå dit hår med den arm, hvor skulderen er problematisk.

Spørgsmål 14

Omhandler det niveau din form var i eller den aktivitet du havde fra før din skulder blev et problem i din nuværende situation. Overvej alle aktiviteter som du føler bidrog til din muskelspænding, styrke eller kondition såsom bowling, fisketure, spadsereture, cykling osv.

Spørgsmål 15

Drejer sig om at nå bagpå for at lyne op eller ned, tage BH af/på, nå din baglomme, klø din ryg osv.

Spørgsmål 16

Drejer sig om ethvert problem med at tage tøj af eller på, at måtte skifte tøjstil eller størrelse på grund af din skulder, eller måtte ændre på den måde du af- eller påklæder dig på grund af din skulder.

**Afsnit D: Følelser**

Spørgsmål 17

Drejer sig om enhver frustration din skulder har voldt dig. Det kan være frustration over dine fysiske begrænsninger eller økonomiske situation alt sammen relateret til dit skulderproblem.

Spørgsmål 18

Drejer sig om bekymringer over at din skulder bliver værre i stedet for bedre eller uforandret.

Spørgmål 19

Drejer sig om følelsen af, at du ulejliger andre med at hjælpe dig med dagligdags opgaver eller ting som du tidligere var vant til selv at gøre.

**SCORING FOR QUALITY OF LIFE MEASUREMENT TOOL FOR OSTEOARTHRITIS OF THE SHOULDER (WOOS)**

1. Measure the distance from the left side of the line and calculate the score out of 100 (recorded to the nearest 0.5 mm.). Write it into the space provided for that question.
2. You can calculate a total score for each domain (Physical Symptoms/600; Sports and Recreation/Work/500; Lifestyle/500;Emotions/300) or the total score for the domains can be summed for an aggregate score out of 1900.
3. Some find it more meaningful to report scores out of 100 i.e. a percentage of normal score. Since is the worst possible score is 1900, the aggregate score is subtracted from 1900 and divided by 19. e.g. total aggregate score = 1625; 1900 - 1625 = 275 / 19 = 14.5%

physical symptoms sports/recreation/work lifestyle emotions

PS1 ________ S 7 __________ L12 _________ E17 __________

PS2 ________ S 8 __________ L13 _________ E18 __________

PS3 ________ S 9 __________ L14 _________ E19 __________

PS4 ________ S10 __________ L15 _________ TOTAL

PS5 ________ S11 __________ L16 _________ ____________

PS6 ________ TOTAL TOTAL

TOTAL ____________ __________

___________

summary

PS ____________

S _____________

L _____________

E _____________

TOTAL

_______________
