# Supplementary material for: Early migration of stemless and stemmed humeral components after total shoulder arthroplasty for osteoarthritis—study protocol for a randomized controlled trial
Source: Trials. 2020 Oct 7;21:830. doi: 10.1186/s13063-020-04763-8 (PMC7541322; doi:10.1186/s13063-020-04763-8)
Supplement: Supplementary file 2 — Additional file 2. Oxford shoulder score. [file 13063_2020_4763_MOESM2_ESM.pdf]

Oxford shoulder score

Patient Label

Problemer med din skulder

Dato: ___________________

præop ☐ 6 mdr ☐ 12 mdr ☐ Venstre ☐ Højre ☐

Sæt et ✕ ved hvert spørgsmål!

1. I løbet af de sidste 4 uger …
Hvordan vil du beskrive den værste smerte du har haft *i din skulder*?

Ingen	Mild	Moderat	Svær	Uudholdelig
☐	☐	☐	☐	☐

2. I løbet af de sidste 4 uger …
Har du haft svært ved at tage tøj på, på grund af din skulder?

Intet besvær	Lidt besvær	Besvær	Meget besvær	Umuligt
☐	☐	☐	☐	☐

3. I løbet af de sidste 4 uger …
Har du haft svært ved at komme ind og ud af en bil eller ved at bruge offentlig transport på grund af din skulder?

Intet besvær	Lidt besvær	Besvær	Meget besvær	Umuligt
☐	☐	☐	☐	☐

4. I løbet af de sidste 4 uger …
Har du været i stand til at bruge kniv og gaffel - *på samme tid*?

Ja, let	Lidt besvær	Besvær	Meget besvær	Umuligt
☐	☐	☐	☐	☐

5. I løbet af de sidste 4 uger …
Kunne du *selv* klare de daglige indkøb?

Ja, let	Lidt besvær	Besvær	Meget besvær	Umuligt
☐	☐	☐	☐	☐

6. I løbet af de sidste 4 uger …
Kunne du bære en bakke med en tallerken med mad gennem et lokale?

Ja, let	Lidt besvær	Nogen besvær	Meget besvær	Umuligt
☐	☐	☐	☐	☐

7. I løbet af de sidste 4 uger …
Kunne du børste/rede dit hår med den dårlige arm?

Ja, let	Lidt besvær	Nogen besvær	Meget besvær	Umuligt
☐	☐	☐	☐	☐

8. I løbet af de sidste 4 uger …
Hvordan vil du beskrive den smerte, du normalt har haft i din skulder?

Ingen	Mild	Moderat	Svær	Uudholdelig
☐	☐	☐	☐	☐

9. I løbet af de sidste 4 uger …
Kunne du hænge dit tøj op i en garderobe, *med din dårlige arm*?

Ja, let	Lidt besvær	Nogen besvær	Meget besvær	Umuligt
☐	☐	☐	☐	☐

10. I løbet af de sidste 4 uger …
Har du været i stand til at vaske og tørre dig selv under begge arme?

Ja, let	Lidt besvær	Nogen besvær	Meget besvær	Umuligt
☐	☐	☐	☐	☐

11. I løbet af de sidste 4 uger …
Hvor meget har smerten fra din skulder forstyrret dit normale arbejde (inkl. husligt arbejde)?

Slet ikke	En lille smule	Moderat	Meget	Totalt
☐	☐	☐	☐	☐

12. I løbet af de sidste 4 uger …
Har du været besværet af smerter i din skulder i din seng om natten?

Ingen nætter	1 til 2 nætter	Nogle nætter	De fleste nætter	Hver nat
☐	☐	☐	☐	☐
