# Supplementary material for: Early migration of stemless and stemmed humeral components after total shoulder arthroplasty for osteoarthritis—study protocol for a randomized controlled trial
Source: Trials. 2020 Oct 7;21:830. doi: 10.1186/s13063-020-04763-8 (PMC7541322; doi:10.1186/s13063-020-04763-8)
Supplement: Supplementary file 3 — Additional file 3. Constant Score. [file 13063_2020_4763_MOESM3_ESM.pdf]

FIGURE 4, PAGE 1

Danish version of the Constant Score test protocol.

Constant Score Copenhagen Denmark 2011

(Efter, Constant CR et al. J. Shoulder Elbow Surg ;Arch/April 2008)

Patientdata (label)	Diagnose ____________________ Hø: Ve: ____
Tlf nr.	Kontroldato:

	Præopr.		
	3 måneder		6 måneder
	1 år		___ år

	POINT
**A. Smerte**<br>Angiv den værste grad af smerte du har oplevet i din skulder ved normale dagligdags aktiviteter, de sidste 24 timer. (0-15 point) (marker med streg på linjen)<br>(Point udregnes efter ligningen: 15 – X = point; X er den målte afstand (cm) fra "ingen smerter" til markeringen (brug en lineal). Decimaler rundes op eller ned til nærmeste heltal, eks. 1,4 cm = 1 point og 1,5 cm = 2 point)<br>Ingen smerter |————————————| Værst tænkelige smerter	
**B. Dagligdags aktiviteter,** de næste 4 spørgsmål omhandler dagligdags aktiviteter, som du har oplevet den seneste uge.	
1. Er din nattesøvn forstyrret af din skulder? (0-2 point)<br>(sæt et kryds)<br>(Point er angivet i parentes)<br>☐ Nej (2)<br>☐ Indimellem (1)<br>☐ Ja, hver nat (0)	
2. Hvor meget af dit normale daglige arbejde tillader din skulder dig at udføre? (0-4 point)<br>(marker med en streg på linjen)<br>(Point udregnes ved at måle afstanden (cm) fra "alt" til markeringen (brug en lineal)):<br>0-3 = 4 point, >3-6 = 3 point, >6-9 = 2 point, >9-12 = 1 point, >12-15 = 0 point<br>Alt |————————————| Intet	
3.Hvor meget af dine normale fritidsaktiviteter tillader din skulder dig at udføre? (0-4 point)<br>(marker med en streg på linjen)<br>(Point udregnes ved at måle afstanden (cm) fra "alt" til markeringen (brug en lineal)):<br>0-3 = 4 point, >3-6 = 3 point, >6-9 = 2 point, >9-12 = 1 point, >12-15 = 0 point<br>Alt |————————————| Intet	
4. Op til hvilken højde kan du bruge din hånd uden ubehag?<br>(0-10 point)(sæt et kryds)<br>(Point er angivet i parentes)<br>☐ Under taljen (0)<br>☐ Op til taljen (2)<br>☐ Op til brystbenet (4)<br>☐ Op til halsen (6)<br>☐ Op til toppen af hovedet (8)<br>☐ Over hovedet (10)	
Samlet score for A+B (subjektive delmål)	

Ilija Ban,[1] Anders Troelsen,[1] David Høyrup Christiansen,[2] Susanne Wulff Svendsen[2] og Morten Tange Kristensen[1,3]
[1]Ortopædkirurgisk Afdeling, Hvidovre Hospital, [2]Dansk Ramazzini Center, Arbejdsmedicinsk Klinik, Regionshospitalet Herning [3]Fysioterapien, Hvidovre Hospital. Dansk oversættelse, Oktober 2011.

FIGURE 4, PAGE 2

Danish version of the Constant Score test protocol.

Constant Score Copenhagen Denmark 2011

(Efter, Constant CR et al. J. Shoulder Elbow Surg ;Arch/April 2008)

C. Bevægelse — POINT

Fire forskellige aktive og smertefrie bevægelser af armen udføres. Dvs. Kan armen løftes til 140 grader med smerte og 110 grader uden smerte i 1+2 så registreres et bevægeudslag på 110 grader.

Tester viser først den ønskede bevægelse, hvorefter testpersonen udfører den. Alle øvelser laves med testpersonen stående med en skulder breddes afstand mellem fødder, der skal pege lige fremad.

1+2

Fleksion og abduktion registreres med et langt goniometer. Bevægelserne udføres kun på skadet side. (0-20 point)

Reference punkterne er armens akse og procesus spinosi af columna thoracalis.

	0-30	31-60	61-90	91-120	121-150	151 -	Bevægeudslag (grader)
Fleksion							
Abduktion							
	0	2	4	6	8	10	Point

3

Udadrotation udføres uden hjælp og hånden skal placeres bag og over hovedet uden at hånden rører hovedet.
(0-10 point)
Bevægelserne udføres med begge arme samtidigt men registreres kun på skadet side, startende med "hånden bag hovedet, albuen frem"

Bevægelserne skal udføres smertefrit.
(2 point gives for hver separat udført bevægelse)

☐	Hånd bag hovedet, albuen frem.
☐	Hånd bag hovedet, albuen tilbage
☐	Hånd til toppen af hovedet, albuen frem
☐	Hånd til toppen af hovedet, albuen tilbage
☐	Fuld elevation af armen

4

Indadrotation udføres uden hjælp og testpersonen bruger sin tommelfinger til at pege på de anatomiske punkter angivet til højre.
(0-10 point)
Bevægelserne udføres kun på skadet side, startende med "ydersiden af låret".

Bevægelserne skal udføres smertefrit.
(point er anført i parentes)

☐	Yderside af lår (0)
☐	Ballen (2)
☐	Sacroiliaca leddet (4)
☐	Taljen (6)
☐	12. Toracale hvivel (8)
☐	Mellem skulderbladene (10)

D Styrke (0-25 point)

Styrken måles med et dynamometer. Testpersonen skal være stående med en skulder breddes afstand mellem fødderne, der skal pege lige fremad. Armen skal være abduceret 90 grader i scapulas plan. Kan armen ikke eleveres til 90 grader gives 0 point. Håndleddet proneres så håndfladen vender ned ad og albuen strækkes mest muligt. Stroppen skal placeres omkring håndleddet på testpersonen så den ligger henover caput af ulna.
Testpersonen instrueres i at presse maksimalt opad i 5 sekunder.
Samtidigt gives verbal opmuntring: Klar 3-2-1 pres..pres..pres

Scoren beregnes ud fra den højeste score af 3 forsøg, hver udført med mindst 1 minuts mellemrum. Scoren svarer til styrken i pund (max 25 point). Måles styrken i kilogram udregnes scoren ved at gange med 2.2

	1. forsøg	2. forsøg	3.forsøg	Bedste score
Styrke (pund/kg)				

Samlet score for C+D (objektive delmål)

Samlet Constant Score A+B+C+D

Ilija Ban,[1] Anders Troelsen,[1] David Høyrup Christiansen,[2] Susanne Wulff Svendsen[2] og Morten Tange Kristensen[1,3]
[1]Ortopædkirurgisk Afdeling, Hvidovre Hospital, [2]Dansk Ramazzini Center, Arbejdsmedicinsk Klinik, Regionshospitalet Herning [3]Fysioterapien, Hvidovre Hospital. Dansk oversættelse, Oktober 2011.
