# Supplementary material for: Early migration of stemless and stemmed humeral components after total shoulder arthroplasty for osteoarthritis—study protocol for a randomized controlled trial
Source: Trials. 2020 Oct 7;21:830. doi: 10.1186/s13063-020-04763-8 (PMC7541322; doi:10.1186/s13063-020-04763-8)
Supplement: Supplementary file 4 — Additional file 4. External assessor. [file 13063_2020_4763_MOESM4_ESM.docx]

**External assessor**

Name: Kristoffer Weisskirchner Barfod

Position: Senior Researcher, Junior Consultant, PhD, MD

Workplace: Hvidovre Hospital

E-mail: [kristoffer.weisskirchner.barfod.02@regionh.dk](mailto:kristoffer.weisskirchner.barfod.02@regionh.dk)
