# Supplementary material for: Early migration of stemless and stemmed humeral components after total shoulder arthroplasty for osteoarthritis—study protocol for a randomized controlled trial
Source: Trials. 2020 Oct 7;21:830. doi: 10.1186/s13063-020-04763-8 (PMC7541322; doi:10.1186/s13063-020-04763-8)
Supplement: Supplementary file 5 — Additional file 5. Informed consent to participate in the health science research project. [file 13063_2020_4763_MOESM5_ESM.docx]

**Informeret samtykke til deltagelse i det sundhedsvidenskabelige forskningsprojekt:**

Early Migration of Stemless and Stemmed Humeral Components After Total Shoulder Arthroplasty for Osteoarthritis – A Randomized Controlled Trial

Version 1.4, 10.10.2019

**Erklæring fra forsøgspersonen:**

Jeg har fået skriftlig og mundtlig information og jeg ved nok om formål, metode, fordele og ulemper til at sige ja til at deltage.

Jeg ved, at det er frivilligt at deltage, og at jeg altid kan trække mit samtykke tilbage uden at miste mine nuværende eller fremtidige rettigheder til behandling.

Jeg giver samtykke til at deltage i forskningsprojektet og har fået en kopi af dette samtykkeark, samt en kopi af den skriftlige information om projektet til eget brug.

Forsøgspersonens navn:

Dato: Underskrift:

**Erklæring fra den der afgiver information:**

Jeg erklærer, at forsøgspersonen har modtaget mundtlig og skriftlig information om forsøget.

Efter min overbevisning er der givet tilstrækkelig information til, at der kan træffes beslutning om deltagelse i forsøget.

Navnet på den, der har afgivet information:

Dato: Underskrift:
