# Supplementary material for: Early migration of stemless and stemmed humeral components after total shoulder arthroplasty for osteoarthritis—study protocol for a randomized controlled trial
Source: Trials. 2020 Oct 7;21:830. doi: 10.1186/s13063-020-04763-8 (PMC7541322; doi:10.1186/s13063-020-04763-8)
Supplement: Supplementary file 7 — Additional file 7. Data category and information. [file 13063_2020_4763_MOESM7_ESM.docx]

| **Data category** | **Information** |
| --- | --- |
| 1. Primary registry and trial identifying number | ClinicalTrials.gov NCT04105478 |
| 1. Date of registration in primary registry | 25 September, 2019 |
| 1. Secondary identifying numbers | H-20017412 - H-19053566 (The Danish National Committee on Health Research Ethics)  MS50976 (Sponsor - Zimmer Biomet, Warsaw, Indiana, United States of America) |
| 1. Source(s) of monetary or material support | Zimmer Biomet, Warsaw, Indiana, United States of America |
| 1. Primary sponsor | Zimmer Biomet, Warsaw, Indiana, United States of America |
| 1. Secondary sponsor(s) | Herlev and Gentofte Hospital, Denmark |
| 1. Contact for public queries | Marc Randall Kristensen Nyring, MD  Herlev og Gentofte Hospital Orthopedic Department Section for Shoulder and Elbow Surgery Opgang 12 Kildegårdsvej 28 2900 Hellerup  Denmark  Phone: +45 51889121  Email: marc.randall.kristensen.nyring@regionh.dk |
| 1. Contact for scientific queries | Bo Sanderhoff Olsen, professor, PhD  Herlev og Gentofte Hospital Orthopedic Department Section for Shoulder and Elbow Surgery Opgang 12 Kildegårdsvej 28 2900 Hellerup  Denmark  Phone: +45 38673280  Email: bo.sanderhoff.olsen@regionh.dk |
| 1. Public title | A Comparison of Early Micro Movements Between Stemless and Stemmed Shoulder Arthroplasties – Study Protocol for a Randomized Controlled Trial |
| 1. Scientific title | Early Migration of Stemless and Stemmed Humeral Components After Total Shoulder Arthroplasty for Osteoarthritis – Study Protocol for a Randomized Controlled Trial |
| 1. Countries of recruitment | Denmark |
| 1. Health condition(s) or problem(s) studied | Glenohumeral osteoarthritis |
| 1. Intervention(s) | Intervention group: Comprehensive Nano stemless total shoulder arthroplasty (Zimmer Biomet, Warsaw, Indiana, United States of America)  Control group: Comprehensive stemmed total shoulder arthroplasty (Zimmer Biomet, Warsaw, Indiana, United States of America) |
| 1. Key inclusion and exclusion criteria | Inclusion Criteria:   1. Primary glenohumeral osteoarthritis independent of previous joint preserving surgery 2. Osteoarthritis on plain radiographs with standard anterior-posterior and lateral projections 3. Insufficient effect of non-surgical treatment with symptoms severe enough to justify shoulder arthroplasty. 4. ASA score 1-3, physically fit for surgery and rehabilitation   Exclusion criteria:   1. Below 18 years of age 2. Cognitive or linguistic impairment 3. Rotator cuff insufficiency defined as rotator cuff lesions or grade 2 fat infiltrations on MRI verified with impaired functional strength and perioperative findings 4. Insufficient glenoid bone-stock or large (> 1cm) humeral bone cysts on CT verified with perioperative findings. 5. ASA score 4-5 |
| 1. Study type | Interventional Allocation: Randomized Intervention model: Parallel 1:1 assignment Masking: Unmasked Primary purpose: Optimizing the treatment of glenohumeral osteoarthritis |
| 1. Date of first enrolment | March 1, 2020 |
| 1. Target sample size | 122 |
| 1. Recruitment status | Recruiting |
| 1. Primary outcome(s) | Outcome name: Magnitude of migration of the humeral component  Method of measurement: Model-based radiostereometric analysis (MB-RSA)  Timepoint: 2 years follow-up  Outcome name: Patient-reported outcome  Method of measurement: Western Ontario Osteoarthritis of the Shoulder index (WOOS)  Timepoint: 2 years follow-up |
| 1. Key secondary outcomes | Outcome name: Bone mineral density  Method of measurement: Duel energy x-ray absorptiometry  Timepoint: 2 years follow-up  Outcome name: Patient-reported outcome  Method of measurement: Oxford Shoulder Score  Timepoint: 2 years follow-up  Outcome name: Combined subjective and objective shoulder scoring system  Method of measurement: Constant-Murley Score  Timepoint: 2 years follow-up  Outcome name: Pain  Method of measurement: Visual analogue scale (VAS)  Timepoint: 2 years follow-up |
