# Supplementary material for: Early migration of stemless and stemmed humeral components after total shoulder arthroplasty for osteoarthritis—study protocol for a randomized controlled trial
Source: Trials. 2020 Oct 7;21:830. doi: 10.1186/s13063-020-04763-8 (PMC7541322; doi:10.1186/s13063-020-04763-8)
Supplement: Supplementary file 8 — Additional file 8. Justification for criteria left as N/A. [file 13063_2020_4763_MOESM8_ESM.docx]

**Justification for criteria left as N/A**

11b:

Since the intervention is performed only once and is not a continuous intervention, this point is not relevant.

11c:

Since the intervention is performed only once and is not a continuous intervention, this point is not relevant.

17b:

The trial is unmasked.

30:

There are no provisions for post-trial care.
